# Results from a multicenter, randomized, double‐blind, placebo‐controlled study of repository corticotropin injection for multiple sclerosis relapse that did not adequately respond to corticosteroids

**DOI:** 10.1111/cns.13789

**Published:** 2022-01-04

**Authors:** Daniel Wynn, Lawrence Goldstick, William Bauer, Enxu Zhao, Eva Tarau, Jeffrey A. Cohen, Derrick Robertson, Aaron Miller

**Affiliations:** ^1^ Consultants in Neurology Multiple Sclerosis Center Northbrook Illinois USA; ^2^ University of Cincinnati Waddell Center for Multiple Sclerosis Cincinnati Ohio USA; ^3^ Department of Neurosciences University of Toledo Toledo Ohio USA; ^4^ Mallinckrodt Pharmaceuticals Hampton New Jersey USA; ^5^ Mellen MS Center Cleveland Clinic Cleveland Ohio USA; ^6^ Department of Neurology University of South Florida Tampa Florida USA; ^7^ Icahn School of Medicine at Mount Sinai Hospital New York New York USA

**Keywords:** Acthar Gel, clinical trial, multiple sclerosis, relapse, repository corticotropin injection

## Abstract

**Introduction:**

About 20%–35% of multiple sclerosis (MS) patients fail to respond to high‐dose corticosteroids during a relapse. Repository corticotropin injection (RCI, Acthar^®^ Gel) is a naturally sourced complex mixture of adrenocorticotropic hormone analogs and pituitary peptides that has anti‐inflammatory and immunomodulatory effects.

**Aims:**

The study objective was to determine the efficacy and safety of RCI in patients with MS relapse that inadequately responded to corticosteroids. This was a multicenter, double‐blind, placebo‐controlled study. Nonresponders to high‐dose corticosteroids were randomized to receive RCI (80 U) or placebo daily for 14 days. Assessments included improvements on the Expanded Disability Status Scale (EDSS), Multiple Sclerosis Impact Scale (MSIS‐29), Clinical Global Impression of Improvement (CGI‐I), and adverse events (AEs).

**Results:**

Eighteen patients received RCI, and 17 received placebo. A greater proportion of EDSS responders was observed in the RCI group at Day 7, 21, and 42 compared with the placebo group. Qualitative CGI‐I showed that more patients receiving RCI were *much improved* or *very much improved* than with placebo. No meaningful differences were observed between treatment groups for MSIS‐29. No serious AEs or deaths were reported.

**Conclusion:**

RCI is safe and effective for MS relapse patients who do not respond to high‐dose corticosteroids.

## Graphical abstract

This study evaluates repository corticosteroid injection (RCI) for treating patients with refractory multiple sclerosis (MS) relapse. A greater proportion of patients were Expanded Disability Status Scale (EDSS) responders after treatment with RCI than with placebo at Days 7, 21, and 42. These findings suggest that RCI is effective for patients with MS relapse that does not respond to high‐dose corticosteroids.

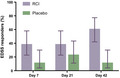

## 1 INTRODUCTION

Relapsing‐remitting multiple sclerosis (RRMS) is a complex disease affecting the central nervous system with several pathophysiological mechanisms such as inflammation, demyelination, axonal damage, and repair.^1^ Relapses are considered to be a hallmark of RRMS, which is the most common form of multiple sclerosis (MS).^2^ In clinical trials, a relapse is normally defined as a minimum symptom period of 24–48 h accompanied by changes in functional measures.^2^ MS relapses often result in significant functional impairments and decreased quality of life.^2^, ^3^ A relapse is usually followed by a period of remission, but incomplete recovery and residual symptoms can persist, leading to worsening disability.^2^, ^4^ Common triggers for relapses include infection and stress.^2^, ^4^ Relapses may reflect new demyelination or reactivation of previous demyelinated lesions in the central nervous system.^2^

Disease‐modifying therapies are highly effective, but relapses still occur and require treatment to prevent persistent residual symptoms and disability.^2^, ^5^ The standard of care therapy for relapses is high‐dose corticosteroids.^2^, ^6^ However, it is estimated that 20%–35% of patients with MS do not respond to high‐dose corticosteroids,^2^ so alternative therapies are needed for these patients with refractory illness.

Repository corticotropin injection (RCI; Acthar^®^ Gel, Mallinckrodt Pharmaceuticals, Hampton, NJ, USA) is a naturally sourced complex mixture of adrenocorticotropic hormone (ACTH) analogs and other pituitary peptides.^7^ RCI is currently approved by the US Food and Drug Administration (FDA) for treatment of exacerbations of MS.^7^ RCI is anti‐inflammatory by stimulating endogenous corticosteroid production and can exert additional immunomodulatory effects by binding to melanocortin receptors (MCRs) on immune cells.^8^, ^9^, ^10^ Notably, in vitro studies have shown that RCI elicits distinct functional activity at MCRs from that of synthetic MCR agonists.^8^ MC1R, MC3R, MC4R, and MC5R are expressed in immune cells and other cell types throughout the body.^6^ MC2R is expressed in adrenocortical cells and upon activation stimulates the production of endogenous cortisol.^6^ Synthetic ACTH_1‐24_ has its highest activity at MC2R, while RCI has its lowest full agonistic activity at MC2R.^8^ Consistent with their MC2R activity profiles, studies in animals and in healthy human subjects have demonstrated much lower endogenous cortisol production with RCI than with synthetic ACTH_1‐24_ depot.^8^, ^11^ This suggests that RCI primarily functions through direct modulation of immune cells, rather than through endogenous cortisol production from the adrenal cortex.^8^ RCI has been shown to be immunomodulatory because it inhibits B‐cell proliferation, antibody production, and inflammatory cytokine production from T cells and macrophages.^10^, ^12^, ^13^

The objective of this clinical trial was to determine the efficacy and safety of RCI for treatment of relapses in patients with RRMS that failed to respond to high‐dose corticosteroids.

## 2 METHODS

### 2.1 Study design

This was a multicenter, randomized, double‐blind, placebo‐controlled study to estimate the response rate and examine the safety of RCI in patients with RRMS who had inadequate responses to high‐dose intravenous methylprednisolone (IVMP), oral prednisone, or oral methylprednisolone. The study was conducted across 31 centers in the United States of America from 2017 to 2020, in accordance with the principles and requirements of the Declaration of Helsinki, Good Clinical Practices, and clinical trial registration (ClinicalTrials.gov identifier NCT03126760). All participating patients provided written informed consent prior to enrollment in the study.

### 2.2 Study population

Patients with RRMS who experienced a relapse with onset ≤42 days prior to the baseline visit and received 3 to 5 days (given over a period of up to 7 days) of treatment with high‐dose IVMP (1000 mg /day), oral prednisone (1250 mg per day [QD]), or oral methylprednisolone (1000 mg QD) within 28 days of the onset of relapse symptoms were eligible to participate in the study. All patients had no prior use of RCI. A screening visit took place in the initial 28 days of the 42 days screening period, and patients were assessed with the Expanded Disability Index Scale (EDSS)/Function Systems Score (FSS) prior to treatment with IVMP, oral prednisone, or oral methylprednisolone. At 14 ± 1 days following the initiation of high‐dose corticosteroids, patients were reassessed with EDSS/FSS and were defined as nonresponders (eligible for inclusion) if they did not improve by at least 1 point in one or more functions of the FSS. Other key inclusion criteria were being adult males or nonpregnant females with a diagnosis of RRMS, as well as having an EDSS score of 2.0–6.5 at the baseline visit and Hemoglobin A1C (HbA1c) ≤6.5% at screening.

Patients were ineligible if they had known contraindications to RCI or known sensitivity to ACTH preparations or to porcine protein products. Patients were excluded if they only had sensory, bowel/bladder, and/or cognitive symptoms of MS associated with the most recent relapse. Those who were receiving any disease‐modifying treatments must have been on a stable dose(s) for 30 days prior to the baseline visit and plan to remain on that dose(s) throughout the study. Patients were excluded from the clinical trial if they were treated with daclizumab or any immunosuppressants in the 6 months prior to the screening visit or throughout the study.

### 2.3 Interventions and assessment schedule

**FIGURE 1:**
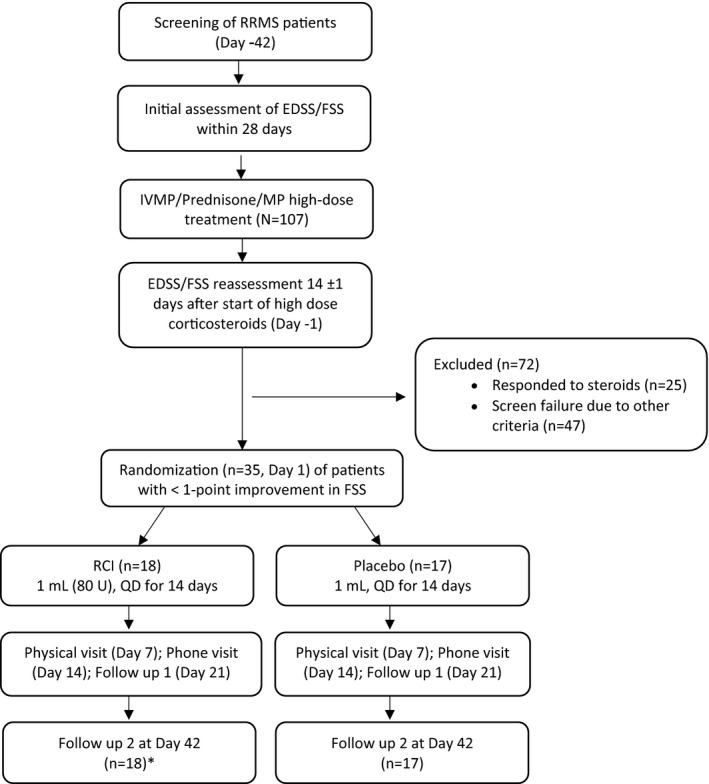
Study flow diagram. *One patient withdrew after 8 treatments but returned at final follow‐up visit. Abbreviations: EDSS, Expanded Disability Status Scale; FSS, function system score; IVMP, intravenous methylprednisolone; MP, methylprednisolone; QD, daily dose; RCI, repository corticotropin injection; RRMS, relapsing‐remitting multiple sclerosis

Eligible patients were randomized according to a computer‐generated allocation scheme to receive either RCI 1 mL (80 U) or placebo 1 mL QD for 14 consecutive days. Treatment response was evaluated using EDSS and other measures up to 42 days after study drug dosing. Patients participated in the study for up to 13 weeks, including a screening period of up to 42 days and an active treatment period of 14 days, and follow‐up visits occurred at 21 ± 2 days and 42 ± 2 days after the start of study drug administration. The study design and treatments are summarized in Figure 1.

### 2.4 Efficacy assessments

The primary objective of this study was to determine the EDSS response rate with 90% confidence intervals (CIs) at Day 42 for each treatment group. Other assessments included mean scores and 90% CIs for MSIS‐29 response rates at Days 7, 14, 21, and 42; the EDSS response rates at Days 7 and 21; and Clinical Global Impression of Improvement Scale (CGI‐I) on Days 7, 21, and 42 for each treatment group. Quality of life was determined by work productivity and activity impairment (WPAI) scores, health care resource utilization (HRU), functional system score (FSS), and Centre for Disease Control (CDC) health‐related quality of life‐4 (HRQOL‐4) self‐assessment.

### 2.5 Safety outcomes

At the screening visit, both prior and current medical conditions were recorded, as well as the last date of the menstrual period in female patients. Prior to administration of the drug, all women of childbearing age must have had a negative pregnancy test. Physical examinations, clinical laboratory tests, pregnancy testing, medical history, weight, and vital signs were monitored at screening and throughout the study. Adverse events (AEs) were recorded and followed by the investigator until the AE had resolved or stabilized, with the first assessment made at the screening visit after treatment with corticosteroids and before treatment with RCI. Adverse events of special interest (AESIs) were elevated blood pressure, hyperglycemia, and AEs considered possibly, probably, or definitely related to study drug treatment of greater than moderate intensity or any infection/infestation as defined by Medical Dictionary for Regulatory Activities (MedDRA) System Organ Class as being greater than moderate intensity and Hy's Law cases. Treatment‐emergent adverse events (TEAEs) were defined as AEs that started or worsened on or after the first dose of the study drug. Treatment‐related AEs were those TEAEs that were considered possibly related or related to the test drug.

### 2.6 Statistical analysis

The primary analysis was to generate point estimates and associated 90% CIs for EDSS response rates in the RCI group and the placebo group at Day 42 using the modified intention‐to‐treat (mITT) population. This study planned to enroll a total of 66 patients randomized in a 1:1 ratio to either RCI or placebo (33 per group). It was assumed that 3 patients per treatment group would not qualify for the mITT population after randomization in each treatment group, so there would be 30 patients in each group. Based on an expected 60% response rate in the RCI group, the study could build a 90% Wilson CI of 45.1% and 73.3% with an approximate precision of 14.1%. Based on an expected 25% response rate in the placebo group, the study could build an approximate 90% Wilson CI of 15.7% and 41.5% with an approximate precision of 12.9%.

The study was terminated early by sponsor decision because of recruitment difficulties and the impact of the coronavirus disease 2019 (COVID‐19) pandemic. The planned analyses did not change except that the total sample size was reduced from 66 to 35. Inferential statistics were not performed. Results are nominal because the sample size did not reach that specified in the protocol.

## 3 RESULTS

### 3.1 Patient selection, demographics, and characteristics

From the screening of 107 RRMS patients, there were 72 total screen failures; 25 responded to high‐dose steroid therapy, while the remaining 47 did not respond to high‐dose steroid therapy, but failed screening due to other criteria such as laboratory abnormalities (ie, HbA1c elevation, anemia, or aspartate aminotransferase/alanine aminotransferase elevation poststeroids). Specific steroid use was not captured in these patients; however, based on randomized patient data, it can be assumed that approximately 35 (75%) of these patients did not respond to IVMP and 12 (25%) did not respond to oral prednisone.

A total of 35 patients were randomized, with 18 patients assigned to the RCI arm and 17 patients to the placebo arm (Figure 1). One patient from the RCI group withdrew after 8 treatments because of TEAEs of edema and pain. However, the patient returned to complete the final visit.

Baseline demographics and patient characteristics are summarized in Table 1. The 2 treatments groups were generally comparable for age, sex, race, ethnicity, weight, height, body mass index (BMI), and baseline EDSS. In this study, 74.3% of participants were between 21 and 45 years old. Most patients were White, non‐Hispanic/Latino women.

**TABLE 1:** Patient demographics and baseline characteristics

Demographic Characteristic	RCI (n = 18)	Placebo (n = 17)
Age (years)		
Mean	41.4	41.2
SD	10.40	12.04
Min, max	21, 62	26, 64
Age category (years)		
≤35	4 (22.2)	6 (35.3)
36–45	10 (55.6)	6 (35.3)
46–55	2 (11.1)	2 (11.8)
56–65	2 (11.1)	3 (17.6)
66–75	0	0
>75	0	0
Sex, n (%)		
Male	3 (16.7)	5 (29.4)
Female	15 (83.3)	12 (70.6)
Race, n (%)		
American Indian or Alaska Native	0	0
Asian	0	0
Black or African American	3 (16.7)	2 (11.8)
Native Hawaiian or other	0	0
White	15 (83.3)	15 (88.2)
Other	0	0
Ethnicity, n (%)		
Hispanic or Latino	0	1 (5.9)
Not Hispanic or Latino	17 (94.4)	16 (94.1)
Not reported	0	0
Unknown	1 (5.6)	0
Weight at baseline (kg)		
Mean	77.52	83.85
SD	17.91	24.54
Min, max	55.0, 107.3	58.1, 134.3
Height (cm)		
Mean	171.09	169.68
SD	8.66	9.71
Min, max	152.4, 190.5	158.8, 188.0
BMI at baseline (kg/m^2^)		
Mean	26.43	29.25
SD	5.57	8.71
Min, max	19.4, 34.6	20.7, 45.0
Baseline EDSS Score		
Mean	3.86	3.85
SD	1.12	1.17
Min, max	2.0, 6.0	2.0, 6.5

Note

Percentages are based on the number of patients in each column header. Age is calculated relative to informed consent date.

Abbreviations: BMI, body mass index; EDSS, Expanded Disability Status Scale; RCI, repository corticotropin injection; SD, standard deviation.

### 3.2 Efficacy

**FIGURE 2:**
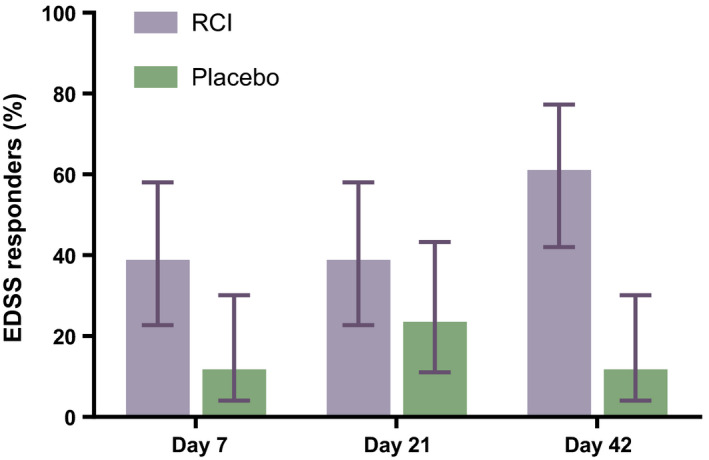
Proportion of EDSS responders for each treatment group. Responders are defined as a subject who achieved a ≥1.0‐point improvement in EDSS score compared with baseline if the baseline EDSS score was ≤5.5 or a ≥0.5‐point improvement in EDSS score compared with baseline if the baseline EDSS score was >5.5. Error bars are 90% confidence intervals. Abbreviations: EDSS, Expanded Disability Status Scale; RCI, repository corticotropin injection

**FIGURE 3:**
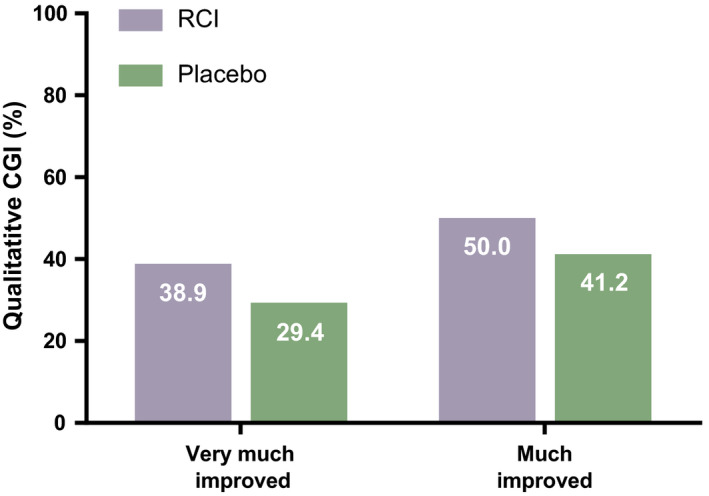
Qualitative CGI‐I scores shown as the proportion of patients who were *very much improved* or *much improved* at Day 42 after treatment. Abbreviations: CGI‐I, Clinical Global Impression of Improvement Scale; RCI, repository corticotropin injection

The primary objective of the study was met, with more EDSS responders in the RCI group vs the placebo group (61.1% [90% CI: 42.0–77.3] vs 11.8% [90% CI: 4.0–30.1], respectively) at Day 42 (Figure 2). The number of EDSS responders was also higher in the RCI group vs placebo group on Days 7 (38.9% [90% CI: 22.7–58.0] vs 11.8% [90% CI: 4.0–30.1] and 21 (38.9% [90% CI: 22.7–58.0] vs 23.5% [90% CI: 11.0–43.3] (Figure 2). MSIS‐29 did not show meaningful differences between treatment groups at any time point (data not shown). Qualitative CGI‐I results showed more patients described as being *much improved* (RCI: 50.0% vs placebo: 41.2%) or *very much improved* (RCI: 38.9% vs placebo: 29.4%) at Day 42 with RCI compared to placebo (Figure 3). There was no meaningful difference in the mean CGI‐I scores between treatment groups (data not shown). Patient‐reported outcomes (PROs) for quality of life also did not show meaningful differences between the RCI and placebo groups (data not shown).

### 3.3 Safety

AEs recorded after corticosteroid treatment but prior to RCI initiation are presented in Table S1. After RCI initiation, the incidence of TEAEs throughout the study was similar between RCI and placebo groups (Table 2). The RCI group had slightly more TEAEs compared to the placebo (77.8% vs 70.6%, respectively). The proportion of patients who experienced TEAEs that were considered by the investigator as possibly related or related to treatment were 61.1% in the RCI group and 47.1% in the placebo group. In the RCI treatment group, 1 patient withdrew from the study due to TEAEs of moderate edema and pain, which resolved 3 weeks after discontinuation and the patient returned for the final follow‐up. The most reported TEAE in both treatment groups was injection site bruising (RCI: 16.7% vs placebo: 23.5%). AESIs were reported for 7 (38.9%) patients in the RCI group and 2 (11.8%) patients in the placebo group. These included generalized edema, pain, nasopharyngitis, oral candidiasis, urinary tract infection, alanine aminotransferase elevation, fluid retention, and insomnia in the RCI group, and injection site inflammation and headache in the placebo group. No specific AESI was reported in more than 1 patient. There were no serious adverse events (SAEs) or deaths.

**TABLE 2:** Treatment‐emergent adverse events (TEAEs)

TEAEs	RCI (n = 18) n (%)	Placebo (n = 17) n (%)
Any TEAE	14 (77.8)	12 (70.6)
Any Mild TEAE	11 (61.1)	12 (70.6)
Any Moderate TEAE	7 (38.9)	5 (29.4)
Any Severe TEAE	0	1 (5.9)
Any Treatment Related TEAE	11 (61.1)	8 (47.1)
Any Serious TEAE	0	0
Any TEAE of Special Interest	7 (38.9)	2 (11.8)
TEAEs leading to Discontinuation of the Study Drug	1 (5.6)	0
Any Serious TEAEs	0	0
Any TEAE leading to Death	0	0
Any Life‐threatening Serious TEAE	0	0
**GENERAL DISORDERS AND ADMINSTRATION SITE CONDITIONS**	6 (33.3)	8 (47.1)
Injection site bruising	3 (16.7)	4 (23.5)
Injection site erythema	1 (5.6)	2 (11.8)
**INJURY, POISONING AND PROCEDURAL COMPLICATIONS**		
Contusion	2 (11.1)	0
**MUSCULOSKELETAL AND CONNECTIVE DISORDERS**	1 (5.6)	5 (29.4)
Arthralgia	1 (5.6)	2 (11.8)
**NERVOUS SYSTEM DISORDERS**	1 (5.6)	4 (23.5)
Headache	0	2 (11.8)
**PSYCHIATRIC DISORDERS**	2 (11.1)	2 (11.8)
Insomnia	2 (11.1)	0

Note

TEAEs are defined as AEs that started or worsened in severity on or after the first dose of study drug. Percentages are based on the number of patients in each column header. System organ classes and MedDRA preferred terms are listed if they occurred in 2 or more patients from either treatment group. For each system organ class and preferred term, subjects are counted only once.

Abbreviations: AE, adverse event; MedDRA, Medical Dictionary for Regulatory Activities; RCI, repository corticotropin injection; TEAE, treatment‐emergent adverse event.

The incidence and types of AEs reported during the high‐dose corticosteroid treatment from Day −14 through Day −1 (just prior to randomization) are presented in Table S1. Overall, 48.6% of subjects experienced AEs during steroid treatment. The most common events (occurring in 2 patients each) were erythema, alanine aminotransferase increase, weight gain, MS relapse, insomnia, and dyspepsia. Most AEs were mild, with only 1 event reported as severe, which was an occurrence of dyspepsia that was resolved with medication.

## 4 DISCUSSION

This study, which sought to determine the efficacy and safety of RCI for the treatment of relapse in patients with RRMS that had inadequate response to high‐dose corticosteroids, met its primary objective with a higher proportion of EDSS responders at Day 42 in the RCI group compared to placebo. There were also more EDSS responders in the RCI group compared to placebo at Days 7 and 21, and qualitative CGI‐I assessments were better in the RCI group compared to placebo at Day 42. The MSIS‐29 scores and quality of life PROs were similar between treatment groups. There were no new safety signals associated with RCI treatment, and no SAEs or deaths were reported. RCI was shown to be well tolerated overall in this subset of RRMS patients.

It was previously demonstrated that the total cortisol‐equivalent exposure of subcutaneous RCI (80 U) given daily for 5 days is equivalent to approximately 30 mg IVMP daily for 5 days.^14^ The equivalent dose of 30 mg of IVMP is considerably lower than what is used clinically (1000 mg) to treat relapses in patients with RRMS,^14^ yet RCI was still effective in the current study. It is estimated that 20%–35% of patients do not achieve an adequate response to corticosteroid treatment or cannot tolerate the side effects.^2^, ^6^ In comparison, only one patient in this study discontinued RCI treatment due to TEAEs.

In the current study, only 25 (23%) of 107 screened patients responded to the high‐dose corticosteroid therapy. This relatively low proportion of responders was likely due to the rigid, objective criteria set for nonresponse, including a <1 point improvement in the FSS after only 14 days following initial high‐dose administration of corticosteroid. Moreover, the patients with RRMS in this study included those with potentially more severe relapse than would be commonly seen in a normal treatment environment. In addition, patients received different (intravenous or oral steroid) treatments, and the relatively small number of patients prevents any generalization regarding the incidence of responders.

The findings in this clinical trial are supported by the results of a recent real‐world registry study of the use of RCI for the treatment of MS relapse.^7^ The MS relapse registry reported efficacy and safety outcomes in 125 patients with acute MS relapse treated with RCI over 24 months.^7^ In the current study, there were more EDSS responders at Days 7, 21, and 42 after RCI initiation, which is consistent with the findings in the MS relapse registry.^7^ In the MS relapse registry study, RCI showed improvements in MSIS‐29 and CGI‐I scores at 2 and 6 months compared to baseline.^7^ In the current study, both RCI and placebo groups showed improvements in the MSIS‐29 and CGI‐I scores compared to baseline, but there were no substantial differences between treatment groups. The duration of the current study was only 42 days, so it is possible that greater differences between treatment groups in MSIS‐29 and CGI‐I scores may have been seen if the study were longer.

This study confirmed the safety of RCI, as most TEAEs were mild in severity and occurred at similar frequencies to the placebo group. The MS relapse registry study reported that only 28% of RCI‐treated patients experienced AEs, which is comparatively lower than the 77.8% who experienced TEAEs in the RCI group in the current study. In a large double‐blind randomized clinical trial (N = 259) for RCI use in rheumatoid arthritis, 3.9% of patients had hyperglycemia or hypertension, suggesting that the incidence of these AEs was relatively low in this population.^15^ AESIs defined for the current study included hyperglycemia and hypertension, but no patients experienced these specific events.

This randomized clinical trial demonstrated that RCI is effective in patients with RRMS that did not adequately respond to high‐dose corticosteroids, which suggests that the mechanism of action of RCI is distinct from that of corticosteroids.^8^, ^10^, ^11^ Although RCI does induce endogenous cortisol production from the adrenal cortex, recent studies in healthy human subjects suggest that these cortisol levels are relatively low following biweekly dosing.^11^ Therefore, it is believed that RCI primarily functions through direct binding to MCRs expressed on neurons, microglia, astrocytes, lymphocytes, monocytes, and macrophages,^6^, ^16^, ^17^ which may account for efficacy in patients who did not respond to high‐dose corticosteroids.

Demyelination is a characteristic feature of RRMS and is driven by inflammation and numerous types of immune cells including macrophages, T cells, and B cells.^18^ MCRs exert anti‐inflammatory effects by reducing proinflammatory cytokines, promoting differentiation of regulatory T cells, reducing proliferation of B cells, and increasing production of anti‐inflammatory cytokines.^18^, ^19^, ^20^, ^21^ RCI has been shown to exert a number of changes that may help promote remyelination of axons.^18^ In RRMS animal models, RCI has been shown to attenuate acute exacerbations of the disease, along with reductions in demyelination of the spinal cord, inflammation, and proliferation of CD4^+^ T cells.^22^ Autoantibodies against myelin help to promote demyelination of axons, and in animal studies of systemic lupus erythematosus, RCI reduced the number of autoantibodies observed, suggesting that RCI could also have similar beneficial effects in RRMS.^18^, ^23^ Furthermore, RCI inhibits the IgG production and proliferation of B cells in vitro, which could be beneficial for the remyelination of axons in RRMS.^10^ RCI has been shown to enhance remyelination in a cuprizone‐induced demyelination animal model of MS.^24^

Limitations of this clinical trial include that the study was terminated prematurely, with approximately 50% (35/66) of patients being enrolled due to recruitment issues and the impact of the COVID‐19 pandemic. Nevertheless, the results demonstrate that RCI was safe and effective in patients with MS relapse. In Europe and North America, there is an increased reluctance by patients with RRMS to participate in placebo‐controlled clinical trials and this might partially account for the low recruitment rate.^25^ Another potential cause of low recruitment is that RRMS can be managed effectively with disease‐modifying therapies, reducing the number of relapses.^5^ Further, patients with relapse are often treated with immunotherapies, which made them ineligible to participate in this study.^5^

## 5 CONCLUSIONS

This multicenter, randomized, double‐blinded, placebo‐controlled study was conducted to assess the effects of RCI in patients with MS relapse that failed to adequately respond to high‐dose corticosteroids. RCI‐treated patients showed substantial improvements on the EDSS and qualitative CGI‐I scales compared with placebo. No new or unexpected safety signals were noted, and there were no SAEs or deaths among those who received RCI or placebo. These results support that RCI is safe and effective for the treatment of patients with MS relapse that did not adequately respond to corticosteroids, suggesting that RCI has a unique anti‐inflammatory mechanism of action.

## CONFLICTS OF INTEREST

**Daniel Wynn**, **MD**, has received speaking and/or consulting fees from Acorda Therapeutics, Avanir Pharmaceuticals, Banner Life, Biogen, EMD Serono, Mapi Pharma, Mallinckrodt Pharmaceuticals, Roche/Genentech, Sanofi Genzyme, and Teva. He has received research support from Acorda Therapeutics, Adamas Pharma, Avanir Pharmaceuticals, Bristol Myers Squibb, Chugai Pharma, EMD Serono, Eisai, Jazz Pharmaceuticals, GW Therapeutics, Mallinckrodt Pharmaceuticals, Mapi Pharma, Mylan, Novartis, Osmotica, Receptos/Celgene, SanBio, Sanofi Genzyme, Sunovion, Teva, TG Therapeutics, and the National Multiple Sclerosis Society. **Lawrence Goldstick**, **MD**, has received consultancy fees from Biogen, Celgene, EMD Serono, Genentech/Roche, Mallinckrodt Pharmaceuticals, Novartis, Teva Pharmaceuticals, and Sanofi Genzyme. He has received speaker fees from Biogen, Genentech/Roche, Acorda Therapeutics, Mallinckrodt Pharmaceuticals, the National Multiple Sclerosis Society, and Sanofi Genzyme. He has also received research support from Biogen, Alkermes, Acorda Therapeutics, Genentech/Roche, Mallinckrodt Pharmaceuticals, Novartis, Eli Lilly, Sunovion, and Sanofi Genzyme. **William Bauer, MD, PhD**, has received consultancy fees from Biogen, Mallinckrodt Pharmaceuticals, and Novartis. **Enxu Zhao**, **MS**, is an employee of Mallinckrodt Pharmaceuticals. **Eva Tarau, MD**, is an employee of Mallinckrodt Pharmaceuticals. **Jeffrey A. Cohen**, **MD**, has received consultancy fees from Biogen, Bristol Myers Squibb, Convelo, Genentech, Janssen, NervGen, Novartis, and PSI; speaker fees from H3 Communications; and compensation for serving as an editor of *Multiple Sclerosis Journal*. **Derrick Robertson**, **MD**, has received consultancy fees from Alexion, Biogen, Bristol Myers Squibb, EMD Serono, Genentech, Greenwich Biosciences, Novartis, Sanofi Genzyme, Teva Neuroscience, and Viela Bio. He has received honoraria or speaker fees from Acorda, Alexion, Biogen, Bristol Myers Squibb, EMD Serono, Genentech, Mallinckrodt Pharmaceuticals, Novartis, Sanofi Genzyme, and Teva Neuroscience; and has received research grant support from Biogen, EMD Serono, Genentech, GW Pharmaceuticals, Janssen, Mallinckrodt Pharmaceuticals, MedDay, MedImmune, Novartis, PCORI, Sanofi Genzyme, SunPharma, and TG Therapeutics. **Aaron Miller**, **MD**, has received consultancy fees from Accordant, Acorda Therapeutics, Alkermes, Biogen, Celgene, EMD Serono, Genentech/Roche, Mallinckrodt Pharmaceuticals, Mapi Pharma, Novartis, and Sanofi Genzyme; and speaker fees from Biogen and Genentech/Roche. He has also received research support from Biogen, Genentech/Roche, Mallinckrodt Pharmaceuticals, MedDay Pharmaceuticals, Novartis, and Sanofi Genzyme.

## AUTHOR CONTRIBUTIONS

All authors contributed to the conception or design of the study, analyzed or interpreted data, and contributed to the writing of the manuscript.

## ETHICAL STATEMENT

The study was conducted across 31 centers in the United States of America from 2017 to 2020, in accordance with the principles and requirements of the Declaration of Helsinki, Good Clinical Practices, and clinical trial registration (ClinicalTrials.gov identifier NCT03126760). All participating patients provided written informed consent prior to enrollment in the study.

## Supporting information

Table S1

## Data Availability

The datasets generated during and/or analyzed during the current study are not publicly available. Individual patient data may be requested if allowed per informed consent and appropriately anonymized. Requests should be sent to Mallinckrodt Pharmaceuticals’ department for Clinical Trial Disclosure and Transparency at clinicaltrials@mnk.com
