# Supplementary material for: Results from a multicenter, randomized, double‐blind, placebo‐controlled study of repository corticotropin injection for multiple sclerosis relapse that did not adequately respond to corticosteroids
Source: CNS Neurosci Ther. 2022 Jan 4;28(3):364–71. doi: 10.1111/cns.13789 (PMC8841301; doi:10.1111/cns.13789)
Supplement: Supplementary file 1 — Table S1 [file CNS-28-364-s001.docx]

**Supporting Information**

**Table S1.**  Adverse Events During Steroid Treatment in Patients Selected for Randomization.

| **AEs Reported During Steroid Treatment** | **Patients Selected for Randomization**  **(n = 35)**  **n (%)** |
| --- | --- |
| Any AE | 17 (48.6) |
| Any Mild AE | 15 (42.9) |
| Any Moderate AE | 8 (22.9) |
| Any Severe AE | 1 (2.9) |
| Any Serious AE | 0 |
| Any AE of Special Interest | 0 |
| Any Life-threatening Serious AE | 0 |
| **INFECTIONS AND INFESTATIONS** |  |
| Nasopharyngitis | 2 (5.71) |
| **SKIN AND SUBCUTANEOUS TISSUE DISORDER** |  |
| Erythema | 2 (5.71) |
| **INVESTIGATIONS** |  |
| Alanine aminotransferase increased | 2 (5.71) |
| Weight increased | 2 (5.71) |
| **NERVOUS SYSTEM DISORDERS** |  |
| MS relapse | 2 (5.71) |
| **PYSCHIATRIC DISORDERS** |  |
| Insomnia | 2 (5.71) |
| **GASTROINTESTINAL DISORDERS** |  |
| Dyspepsia | 2 (5.71) |

Percentages are based on the number of patients in each column header. System

organ classes and MedDRA preferred terms are listed if they occurred in 2 or more

patients from either treatment group. For each system organ class and preferred

term, patients are counted only once.

**Abbreviations:** AE, adverse event; MedDRA, Medical Dictionary for Regulatory Activities;

RCI, repository corticotropin injection
